# Depletion of ATP-Citrate Lyase (ATPCL) Affects Chromosome Integrity Without Altering Histone Acetylation in *Drosophila* Mitotic Cells

**DOI:** 10.3389/fphys.2019.00383

**Published:** 2019-04-04

**Authors:** Patrizia Morciano, Maria Laura Di Giorgio, Antonella Porrazzo, Valerio Licursi, Rodolfo Negri, Yikang Rong, Giovanni Cenci

**Affiliations:** ^1^Dipartimento di Biologia e Biotecnologie “Charles Darwin”, Sapienza – Università di Roma, Rome, Italy; ^2^Istituto Pasteur Italia - Fondazione Cenci Bolognetti, Rome, Italy; ^3^Istituto di Analisi dei Sistemi ed Informatica “Antonio Ruberti”, Consiglio Nazionale delle Ricerche, Rome, Italy; ^4^Istituto di Biologia e Patologia Molecolari (IBPM) del CNR, Rome, Italy; ^5^School of Life Sciences, State Key Laboratory of Biocontrol, Sun Yat-sen University, Guangzhou, China

**Keywords:** citrate lyase, Drosophila chromosomes, histone acetylation, acetyl-CoA, Drosophila

## Abstract

The Citrate Lyase (ACL) is the main cytosolic enzyme that converts the citrate exported from mitochondria by the SLC25A1 carrier in Acetyl Coenzyme A (acetyl-CoA) and oxaloacetate. Acetyl-CoA is a high-energy intermediate common to a large number of metabolic processes including protein acetylation reactions. This renders ACL a key regulator of histone acetylation levels and gene expression in diverse organisms including humans. We have found that depletion of ATPCL, the Drosophila ortholog of human ACL, reduced levels of Acetyl CoA but, unlike its human counterpart, does not affect global histone acetylation and gene expression. Nevertheless, reduced ATPCL levels caused evident, although moderate, mitotic chromosome breakage suggesting that this enzyme plays a partial role in chromosome stability. These defects did not increase upon X-ray irradiation, indicating that they are not dependent on an impairment of DNA repair. Interestingly, depletion of ATPCL drastically increased the frequency of chromosome breaks (CBs) associated to mutations in *scheggia*, which encodes the ortholog of the mitochondrial citrate carrier SLC25A1 that is also required for chromosome integrity and histone acetylation. Our results indicate that ATPCL has a dispensable role in histone acetylation and prevents massive chromosome fragmentation when citrate efflux is altered.

## Introduction

Acetyl coenzyme A (acetyl-CoA) is a high-energy intermediate common to a large number of metabolic processes, including lipogenesis and cholesterogenesis that take place in different intracellular compartments. Acetyl-CoA is also required for protein acetylation reactions, which are important in post-translation modifications of proteins such as histones.

Acetyl coenzyme A may be synthesized in mitochondria and exported to the cytosol. This transport is strictly dependent on the citrate-malate-pyruvate shuttle. For this process, mitochondrial acetyl-CoA is first condensed with oxaloacetate by citrate synthase thus producing citrate and free CoA. Citrate is then exported to the cytosol through the mitochondrial citrate carrier SLC25A1. Here, acetyl-CoA generation is mediated by the activity of the ATP-cytrate lyase (ACL), which catalyzes the ATP-dependent cleavage of mitochondrial-derived citrate into oxaloacetate and acetyl-CoA (Chypre et al., 2012). Finally, the malate dehydrogenase 1, NAD (soluble; MDH1) converts cytosolic oxaloacetate by catalyzing the NADH-dependent synthesis of malate, which is transported back to the mitochondria through SLC25A10, an inorganic phosphate/dicarboxylate antiporter. Given its key role in the production of cytosolic acetyl-CoA, it is not surprising that ACL serves several and crucial functions in mammal metabolism (Wellen et al., 2009; Lee et al., 2014, 2015; Das et al., 2015; Covarrubias et al., 2016; Deb et al., 2017; Chen et al., 2018; White et al., 2018).

Although ACL resides mainly in the endoplasmic reticulum in mammalian cells (Chypre et al., 2012), it can be found also in the nucleus (Wellen et al., 2009). Here, as citrate can diffuse through the nuclear membrane, it produces acetyl-CoA, which is directly required to promote histone acetylation and regulate gene expression in response to growth factor stimulation and during differentiation (Wellen et al., 2009). ACL has been recently shown to be phosphorylated at S455 downstream of ataxia telangiectasia mutated (ATM) and AKT following DNA damage (Sivanand et al., 2017). This phosphorylation is necessary for BRCA1 recruitment and DNA repair by homologous recombination and identifies ACL as a molecular player in the DNA damage response.

A large amount of evidence indicate that ACL is upregulated or activated in several types of cancer (Zaidi et al., 2012b). Cancer cells rely on glucose as major carbon source for *de novo* fatty acid synthesis. Glycolysis generates citrate by tricarboxylic acid (TCA) cycle, the citrate is preferentially exported from mitochondria to cytosol and then cleaved by ACL to produce cytosolic acetyl-CoA, the building block for *de novo* lipid synthesis. Thus, by coupling energy metabolism with fatty acids synthesis, ACL plays a critical role in supporting cancer cell growth. It is not therefore unexpected that inhibition of ACL dramatically suppresses tumor cell proliferation (Zaidi et al., 2012a; Migita et al., 2013, 2014; Gao et al., 2014; Lee et al., 2015; Wang et al., 2017; Granchi, 2018).

Here we show that depletion of ATPCL, the Drosophila ortholog of human ACL, leads to a moderate frequency of chromosome breaks (CBs) indicating that this enzyme is partially required for chromosome stability in mitotic cells. Interestingly, the number of CBs is substantially enhanced when the export of citrate from mitochondria is inhibited suggesting that the ATPCL role on chromosome integrity is essential when cytosolic citrate is limited. We also show that *ATPCL* mutants exhibit decreased levels of Acetyl CoA but this reduction does not affect global histone acetylation and gene expression. This indicates that, unlike its human counterpart, the role of ATPCL in Drosophila histone acetylation could be redundant.

## Results

### The Drosophila ATP Cytrate Lyase

**FIGURE 1:**
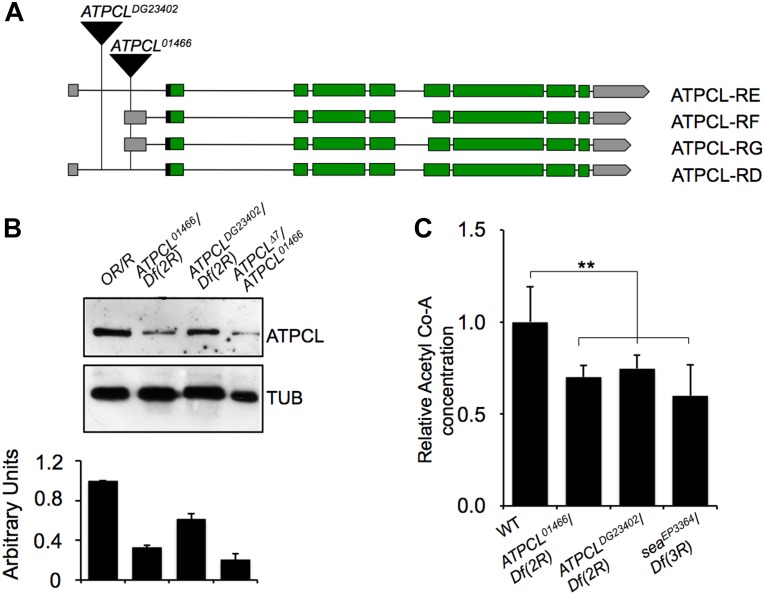
Molecular analysis of *ATPCL* mutations. **(A)** An analysis of the published DNA sequence reveals that ATPCL-encoding gene *CG8322* specifies four potential transcripts RD, RE, RF and RG. The black triangles indicate the position of the *P{PZ}ATPCL^01466^* and *P{wHy}ATPCL^DG23402^* insertions in the *ATPCL* locus referred as *ATPCL^01466^* and *ATPCL^DG23402^*, respectively. Green boxes: encoding exons; gray boxes: UTR-containing exons; horizontal black lines: introns. **(B)** Western blot of third instar larvae brain extracts from control *(OR/R)* and *ATPCL* mutants. The anti-ATPCL antibody recognizes a ∼130 KDa band which is significantly reduced in all *ATPCL* mutant combinations. *Df (2R)* is the *Df(2R)Exel7138* deficiency that removes *ATPCL*. Tubulin is used as a loading control. The bottom figure illustrates the quantification analysis of ATPCL levels, which has been based on band intensities of three independent WBs (biological replicates). Bars indicate standard deviation. **(C)** Reduced levels of Acetyl CoA in *ATPCL* mutants. Columns indicate the percentage of Acetyl-CoA quantification (pmol/ml) in both *ATPCL^DG23402^* and *ATPCL^DG23402^* hemizygotes with respect to OR/R (control; ^∗^*p* < 0.01, Student *t*-test). Bars show SD.

We previously demonstrated that perturbation in the citrate metabolism leads to genome instability in both Drosophila and human cells as consequence of reduced Acetyl-CoA production (Morciano et al., 2009). To further understand the contribution of the acetyl-CoA metabolism to the maintenance of genome stability, we focused our characterization on the ATP Cytrate Lyase. This enzyme cleaves TCA-derived citrate in oxaloacetate OAA (that in turn is converted into malate in order to re-entry in the mitochondria) and catalyzes the formation of acetyl-CoA in the presence of ATP. In *Drosophila*, the citrate lyase-encoding gene (*CG8322*, FBgn0020236) maps on 52D9-11 chromosome 2 region and consists of 10 exons (including the 5′ and 3′ UTR sequences) that, according to the FlyBase annotation, specifies four potential transcripts (namely *ATPCL-RD, -RE, -RG*, and *-RF*) that retain all exons albeit with few differences (Supplementary Text). Our RT-PCR and sequencing analysis confirmed the presence of these different transcripts (Supplementary Text). Moreover, qPCR revealed that *ATPCL-RD* and *RF* are poorly expressed with respect to *ATPCL-RD/RE* suggesting that ATPCL-PD/PE is the most representative *ATPCL* gene product (Figure 1B). Despite the differences in the exons 6, all transcripts encode almost identical ATPCL proteins that overall shares 70% of identity with the human counterpart, hACL. It is therefore conceivable that ATPCL is also functionally analogous to hACL (see also below).

We obtained from the Bloomington stock center two putative P-element induced *ATPCL* mutant alleles, *ATPCL^01466^* and *ATPCL^DG23042^.* Our PCR analysis confirmed that *ATPCL^01466^* and *ATPCL^DG23042^* are located within the 5′ UTR of the gene at position -340 and -1167 upstream of the translation initiation site, respectively (Figure 1A). *ATPCL^01466^* homozygotes and *ATPCL^01466^/ Df(2R)Exel7138* hemizygotes (*Df(2R)Exel7138* is a deficiency in the 52D1-52D12 polytene region that removes *ATPCL*) were late lethal while either *ATPCL^DG23042^* homozygotes and hemizygotes displayed semi-lethality. By mobilizing the *ATPCL^DG23042^* P-element, we generated an additional lethal *ATPCL* mutant allele (Δ7), which potentially bears partial deletions of the gene and severely affected ATPCL expression (see below). This suggests that loss of function of *ATPCL* might prevent development as it occurs also for ACL knock-out mice (Beigneux et al., 2004). Finally, the expression of a wild type *UAS ATPCL* transgene under the control of a *TubGal4* promoter in *ATPCL^DG23042^* or *ATPCL^01466^* mutant background rescued the late lethality and CBs (see section “Materials and Methods”) confirming that both phenotypes are due to lesions in the *ATPCL* gene.

We have generated a guinea pig anti ATPCL polyclonal antibody that in Western blotting (WB) analysis on larval brain extracts detected a band of expected size (∼130 KDa), which decreased in *ATPCL* mutants (Figure 1B). *ATPCL^01466^/ATPCL*^Δ7^ *trans* heterozygotes yielded to the strongest reduction (∼80%) of expression of the ATPCL protein indicating that this mutant allele combination represents the most severe condition (Figure 1B). Immunofluorescence (IF) on larval neuroblasts with the same anti-ATPCL antibody revealed that ATPCL exhibits a punctuate localization pattern both in the cytoplasm and nucleus, which is absent from mutant cells (Supplementary Figure 2). However, as cells enter mitosis, ATPCL is excluded from the portion of nucleus containing chromatin and is retained predominantly in the cytoplasm. This pattern is clearly visible in either metaphase or ana-telophase in which ATPCL is enriched in the entire cell except the portion surrounding the dividing chromosomes (Supplementary Figure 2). Thus, like its human counterpart (Wellen et al., 2009), ATPCL localizes to the cytoplasm and the nucleus in interphase. Moreover, the nuclear localization changes during mitosis.

Finally, our fluorometric analysis revealed that Acetyl-CoA levels are ∼40% reduced in *ATPCL* mutants with respect to wild-type (1.30 pm/μl) indicating that, similarly to its mammalian counterpart, ATPCL is required for Acetyl-CoA synthesis (Figure 1C).

### The ATPCL Role in Mitosis

**FIGURE 2:**
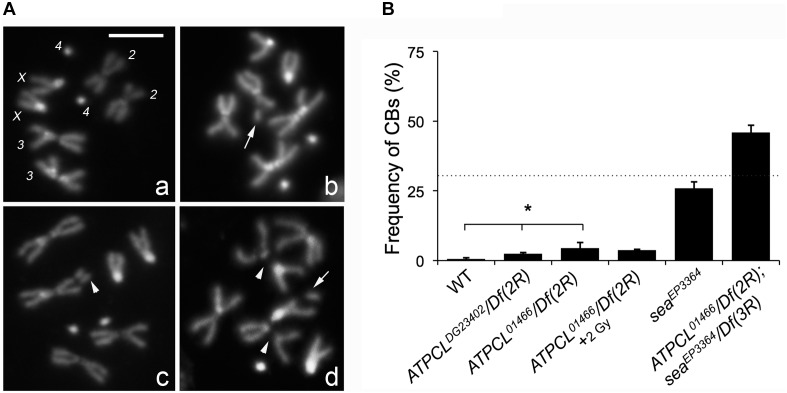
Depletion of ATPCL yields to chromosome breaks (CBs). **(A)** DAPI-stained female metaphases from larval neuroblasts of control (Oregon R, **a**), *ATPCL* mutants **(b,c)** and *ATPCL, sea* double mutants **(d)**. Numbers in **(a)** indicate chromosomes X, 2, 3, and 4 of a normal female caryotype. White arrows and arrowheads indicated chromatid and CBs, respectively. Bar: 5 μm. **(B)** Frequency of CBs in *ATPCL* mutants. All *ATPCL* mutants exhibit a moderated (although statistically significant) frequency of CBs, which are almost absent in control cells (^∗^*p* < 0.05, Student *t*-test). CBs frequency is not influenced by a 2 Gy X-ray irradiation. Note that the frequency of CBs in *ATPCL; sea* double mutants is higher than the sum of CB frequencies observed in either *ATPCL* or *sea* single mutants (indicated by the dotted line) suggesting a synergistic effect. ∼200 cells (4 slides) for mutant combinations and ∼500 (5 slides) for Oregon R (control) were analyzed. See text for further details. Bars refer to SD.

In the citrate-dependent Acetyl CoA biosynthesis, ACL works downstream the citrate mitochondria carrier SLC25A1 (see section “Introduction”). As SLC25A1 and Scheggia (*Sea*, the SLC25A1 fly ortholog) are required for chromosome integrity in mammalian and Drosophila cells, respectively (Morciano et al., 2009), we asked if also ATPCL was involved in the same process. The analysis of DAPI-stained colchicine-treated mitotic neuroblasts from the different *ATPCL* mutant larval brains revealed that *ATPCL* mutants exhibited a moderate, although statistically significant, number of CBs (2.5–4.5%; Total Cells = 250; Figure 2). Moreover 2 Gy X-ray exposure did not enhance the number of CBs of *ATPCL* mutants indicating that they do not result from defective DNA repair (Figure 2B). However, WB and IF analyses using anti-acetylated H4 and H3 histones indicated that the overall histone acetylation in all *ATPCL* mutants was not significantly affected (Supplementary Figure 3). Although it cannot be ruled out the possibility that reduction of histone acetylation occurs at damaged sites, our observations suggest that chromosome defects are not dependent on a general reduction of histone acetylation. We then verified whether *ATPCL* and *scheggia* (*sea*) could genetically interact. The cytological analysis of mitotic metaphases from late lethal *ATPCL sea* double mutants revealed that CBs frequency is synergistically increased (∼46%; Total Cells = 180) with respect to the sum of break frequencies of both *ATPCL* (4.5%) and *sea* ∼25%; (Total Cells = 200; Figure 2B). This indicates that ATPCL prevents genome instability when certain intracellular metabolites (i.e., citrate) are limited.

### ATPCL Is Not Required for Global Gene Transcription

We next asked whether loss of ATPCL could at least influence the expression of potential ATPCL-responsive mitotic genes. Thus we extracted RNA from either wild-type or *ATPCL* mutant brains, labeled, and hybridized to CDMC 14K Drosophila arrays (Canadian Drosophila Microarray Center, Toronto, Canada). Surprisingly, only ∼5% (772) of the genes significantly changed their transcriptional profile indicating that loss of *ATPCL* only marginally affected global gene transcription. Of these, approximately 23% (182) were > 2-fold either down- or up-regulated (Supplementary Table S1). Gene ontology (GO) revealed a clear functional bias toward brain development and behavior gene categories indicating a potential requirement of ATPCL in the regulation of nervous system genes (Supplementary Figure 4). However, based on these results, we cannot discriminate whether the change in gene expression is a direct consequence of the loss of histone acetylation rather than that of secondary responses to metabolic alterations induced by loss of acetyl-CoA. Yet, as microarrays were probed with larval brain RNA we cannot exclude that the observed trends on brain development might reflect a tissue-specific function of ATPCL in gene regulation.

## Discussion

Here we have molecularly characterized for the first time *ATPCL* that encodes the Drosophila ortholog of human ATP citrate lyase. We have shown that, consistently with previous findings in other organisms including mammals, mutations in the *ATPCL* reduce cytosolic acetyl-CoA confirming that ACL has an evolutionarily conserved role in Acetyl-CoA biosynthesis. However, despite *Drosophila ATPCL* mutants exhibit an evident, although moderate, mitotic chromosome breakage phenotype, depletion of ATPCL, unlike human cells, has not a general impact on histone acetylation in flies suggesting that it role in histone acetylation is either partially redundant in Drosophila or compensated by alternative pathways (i.e., from acetate by acyl-CoA synthetases). However, it cannot be ruled out the possibility that ATPCL could be required for the acetylation of either nuclear or cytoplasmic factors, instead of histones, that in turn are required for chromosome integrity. Alternatively, as ATPCL plays a direct role in the homeostasis of lipids, whose modifications (i.e., peroxidation) are also required to modulate DNA repair (Tudek et al., 2017), it is possible that *ATPCL* mutants defects on chromosome integrity could arise as a consequence of protein acetylation-independent activity. Proteomic studies will be fundamental to verify these hypotheses.

We have previously demonstrated that mutations in the mitochondrial citrate carrier Sea/SLC25A1, led to frequent CBs in Drosophila mitotic cells (Morciano et al., 2009). The finding that perturbation of ATPCL, a key component of the pathway that from citrate generates Acetyl-CoA, affected mitosis only marginally was therefore unexpected. Moreover, our WB analysis revealed that, unlike Sea/SLC25A1, ATPCL is not required for the acetylation of histones, which could be required for chromosome integrity. However, this does not exclude that a decreased acetyl-CoA concentration could affect the local kinetics of HAT-dependent histone acetylation at selected genomic loci and/or damaged sites thus leading to specific changes in histone acetylation. Finally, our transcriptomic analysis that indicates that depletion of ATPCL has a modest modulatory effect on transcription, limited to few classes of genes (although with a bias toward brain development functions), further sustains the view that loss of ATPCL has a minimal effect on bulk chromatin histone acetylation.

The different effect of loss of Sea and ATPCL on Drosophila chromosome integrity is intriguing. This is not due to the molecular nature of mutant alleles of either *sea* or *ATPCL*, which are almost genetically null and exhibited a strong reduction of corresponding transcripts. It is conceivable that citrate deprivation in *sea* mutants causes much greater effects than reduction of Acetyl-CoA in *ATPCL* mutants. Indeed, citrate may play different tissue-specific metabolic roles and function as a general chelator of physiologically important cations. Thus its deprivation in the cytosol may compromise several intracellular pathways. Citrate reduction as consequence of inhibition of Sea/SLC25A1 transport activity might also inhibit *ATPCL* activity and/or expression thus exacerbating the *ATPCL* mutant phenotype. It has been recently demonstrated that in Drosophila citrate may indeed regulate a feedback mechanism that coordinates intracellular metabolism with glycolysis and TCA cycle (Li et al., 2018). It could be important to understand whether this metabolic feedback loop may also involve ATPCL. Our findings that *ATPCL* depletion in *sea* mutants yields to a synergic CBs phenotype suggest that ATPCL prevents chromosome fragmentation when citrate metabolism is impaired.

The mild phenotype associated with loss of ATPCL in Drosophila does not preclude the possibility that also the overexpression of ATPCL may impact Drosophila somatic cell proliferation. Overexpression of the human ortholog ACL supports cancer growth by fueling the glucose-dependent *de novo* lipogenesis. Whether a similar histone-acetylation independent effect takes place also in Drosophila will be an interesting issue to address in the future.

## Author Contributions

PM, MDG, and AP performed gene characterization and mutants analysis. VL and RN carried out the microarray analysis. YR supervised gene characterization and discussed the data. GC supervised the experiments and wrote the manuscript.

## Conflict of Interest Statement

The authors declare that the research was conducted in the absence of any commercial or financial relationships that could be construed as a potential conflict of interest.

## References

[B1] Beigneux A. P. Kosinski C. Gavino B. Horton J. D. Skarnes W. C. Young S. G. (2004). ATP-citrate lyase deficiency in the mouse. *J. Biol. Chem.* 279 9557–9564. 10.1074/jbc.M310512200 14662765 PMC2888281

[B2] Chen L. Y. Lotz M. Terkeltaub R. Liu-Bryan R. (2018). Modulation of matrix metabolism by ATP-citrate lyase in articular chondrocytes. *J. Biol. Chem.* 293 12259–12270. 10.1074/jbc.RA118.002261 29929979 PMC6078460

[B3] Chypre M. Zaidi N. Smans K. (2012). ATP-citrate lyase: a mini-review. *Biochem. Biophys. Res. Commun.* 422 1–4. 10.1016/j.bbrc.2012.04.144 22575446

[B4] Covarrubias A. J. Aksoylar H. I. Yu J. Snyder N. W. Worth A. J. Iyer S. S. (2016). Akt-mTORC1 signaling regulates Acly to integrate metabolic input to control of macrophage activation. *eLife* 5:e11612. 10.7554/eLife.11612 26894960 PMC4769166

[B5] Das S. Morvan F. Jourde B. Meier V. Kahle P. Brebbia P. (2015). ATP citrate lyase improves mitochondrial function in skeletal muscle. *Cell Metab.* 21 868–876. 10.1016/j.cmet.2015.05.006 26039450

[B6] Deb D. K. Chen Y. Sun J. Wang Y. Li Y. C. (2017). ATP-citrate lyase is essential for high glucose-induced histone hyperacetylation and fibrogenic gene upregulation in mesangial cells. *Am. J. Physiol. Renal. Physiol.* 313 F423–F429. 10.1152/ajprenal.00029.2017 28490526

[B7] Gao Y. Islam M. S. Tian J. Lui V. W. Xiao D. (2014). Inactivation of ATP citrate lyase by Cucurbitacin B: a bioactive compound from cucumber, inhibits prostate cancer growth. *Cancer Lett.* 349 15–25. 10.1016/j.canlet.2014.03.015 24690568

[B8] Granchi C. (2018). ATP citrate lyase (ACLY) inhibitors: an anti-cancer strategy at the crossroads of glucose and lipid metabolism. *Eur. J. Med. Chem.* 157 1276–1291. 10.1016/j.ejmech.2018.09.001 30195238

[B9] Lee J. H. Jang H. Lee S. M. Lee J. E. Choi J. Kim T. W. (2015). ATP-citrate lyase regulates cellular senescence via an AMPK- and p53-dependent pathway. *FEBS J.* 282 361–371. 10.1111/febs.13139 25367309

[B10] Lee J. V. Carrer A. Shah S. Snyder N. W. Wei S. Venneti S. (2014). Akt-dependent metabolic reprogramming regulates tumor cell histone acetylation. *Cell Metab.* 20 306–319. 10.1016/j.cmet.2014.06.004 24998913 PMC4151270

[B11] Li H. Hurlburt A. J. Tennessen J. M. (2018). A Drosophila model of combined D-2- and L-2-hydroxyglutaric aciduria reveals a mechanism linking mitochondrial citrate export with oncometabolite accumulation. *Dis. Model. Mech.* 11:dmm035337. 10.1242/dmm.035337 30108060 PMC6177012

[B12] Migita T. Okabe S. Ikeda K. Igarashi S. Sugawara S. Tomida A. (2013). Inhibition of ATP citrate lyase induces an anticancer effect via reactive oxygen species: AMPK as a predictive biomarker for therapeutic impact. *Am. J. Pathol.* 182 1800–1810. 10.1016/j.ajpath.2013.01.048 23506848

[B13] Migita T. Okabe S. Ikeda K. Igarashi S. Sugawara S. Tomida A. (2014). Inhibition of ATP citrate lyase induces triglyceride accumulation with altered fatty acid composition in cancer cells. *Int. J. Cancer* 135 37–47. 10.1002/ijc.28652 24310723

[B14] Morciano P. Carrisi C. Capobianco L. Mannini L. Burgio G. Cestra G. (2009). A conserved role for the mitochondrial citrate transporter Sea/SLC25A1 in the maintenance of chromosome integrity. *Hum. Mol. Genet.* 18 4180–4188. 10.1093/hmg/ddp370 19654186

[B15] Sivanand S. Rhoades S. Jiang Q. Lee J. V. Benci J. Zhang J. (2017). Nuclear Acetyl-CoA production by ACLY promotes homologous recombination. *Mol. Cell* 67 252–265.e6. 10.1016/j.molcel.2017.06.008 28689661 PMC5580398

[B16] Tudek B. Zdżalik-Bielecka D. Tudek A. Kosicki K. Fabisiewicz A. Speina E. (2017). Lipid peroxidation in face of DNA damage, DNA repair and other cellular processes. *Free Radic. Biol. Med.* 107 77–89. 10.1016/j.freeradbiomed.2016.11.043 27908783

[B17] Wang D. Yin L. Wei J. Yang Z. Jiang G. (2017). ATP citrate lyase is increased in human breast cancer, depletion of which promotes apoptosis. *Tumour Biol.* 39:1010428317698338. 10.1177/1010428317698338 28443474

[B18] Wellen K. E. Hatzivassiliou G. Sachdeva U. M. Bui T. V. Cross J. R. Thompson C. B. (2009). ATP-citrate lyase links cellular metabolism to histone acetylation. *Science* 324 1076–1080. 10.1126/science.1164097 19461003 PMC2746744

[B19] White P. J. Mcgarrah R. W. Grimsrud P. A. Tso S. C. Yang W. H. Haldeman J. M. (2018). The BCKDH kinase and phosphatase integrate BCAA and lipid metabolism via regulation of ATP-citrate lyase. *Cell Metab.* 27 1281–1293.e7. 10.1016/j.cmet.2018.04.015 29779826 PMC5990471

[B20] Zaidi N. Royaux I. Swinnen J. V. Smans K. (2012a). ATP citrate lyase knockdown induces growth arrest and apoptosis through different cell- and environment-dependent mechanisms. *Mol. Cancer Ther.* 11 1925–1935. 10.1158/1535-7163.MCT-12-0095 22718913

[B21] Zaidi N. Swinnen J. V. Smans K. (2012b). ATP-citrate lyase: a key player in cancer metabolism. *Cancer Res.* 72 3709–3714. 10.1158/0008-5472.CAN-11-4112 22787121

