# Supplementary material for: Depletion of ATP-Citrate Lyase (ATPCL) Affects Chromosome Integrity Without Altering Histone Acetylation in Drosophila Mitotic Cells
Source: Front Physiol. 2019 Apr 4;10:383. doi: 10.3389/fphys.2019.00383 (PMC6458238; doi:10.3389/fphys.2019.00383)
Supplement: Supplementary file 1 [file Data_Sheet_1.PDF]

Depletion of ATP-citrate lyase (ATPCL) affects chromosome integrity without altering histone acetylation in *Drosophila* mitotic cells

Supplementary Material

Supplementary Text

According to FlyBase annotation, the longest *ATPCL-RD* and *-RE* transcripts are identical except for the 3' UTR sequence, which in RE is shorter. *ATPCL-RG* and *RF* transcripts contain and identical 5' UTR sequence which is also different from that of RD and RE. In addition RG, RF and RD contain the same 3' UTR sequence. However, with respect to RE/RD exon 6, exons 6 from RF and RG are lacking of the first 51 and 48 bp, respectively. Thus, the *ATPCL* locus is predicted to encode for 3 different polypetides of 1112 aa (ATPCL-PD/-PE), 1095 aa (ATPCL-PF) and 1096 aa (ATPCL-PG).

The presence of RD, RF and RG transcripts was verified by RT-PCR using specific forward primers designed to include the coding sequence of the adjacent n5 and n6 exons. These primers contained the last 12nt 5'-GTTGGGCTCCGGTTCCG-3' sequence of the exon 5 and either CAG, ATG or CTC 3' nucleotides corresponding to the unique first three bps at the 5' end from exon 6 of RG, RF and RD transcripts, respectively (Supplementary Figure). All forward primers and the reverse primer 5'-ATTCCCCACACAATCGCCTT-3' selected from the same exon 6 sequence (and that is common to all transcripts) were then used to amplify cDNA fragments of predicted 120- (for RG), 117- (for RF) or 168- (for RD) bps (Supplementary Figure). Finally, we found that the sequences of these fragments corresponded to that reported in Flybase and revealed the presence of the CAG, ATG or CTC bps that are unique for each single transcript (Supplementary Figure). The fact that the 3'UTR of RE is included in RG 3'UTR impeded us to design primers for discriminating RE from RD (See Figure 1A).

Materials and Methods

Drosophila strains and crosses.

The *sea*^{EP3364} line was already described (Morciano et al., 2009).The insertion lines *ATPCL*^{01466} and *ATPCL*^{DG23402}, as well as the *Df(2R)Exel7138* that uncovers *ATPCL,* were obtained from the Bloomington Stock Center and were balanced over *CyTb (Lattao et al., 2011;Cipressa et al., 2016)*. *ATPCL* excissants were generated by Delta 2-3-induced incomplete excision of *ATPCL*^{DG23402}. *sea*^{EP3364} double mutant The *ATPCL*^{01466}*/Df(2R)*; was obtained by crossing *ATPCL*^{01466}*(or Df(2R)Exel7138) /Sco; MKRS/TM6b* males to *Sco/CyGFP; sea* ^{EP3364} */TM6B* females. *ATPCL*^{01466}*/ CyGFP*; *sea*^{EP3364}*/TM6b* flies were then crossed to *Df(2R)Exel7138/ CyGFP*; *sea*^{EP3364}*/TM6b* flies. All Drosophila lines were raised on standard corn-meal food.

Acetyl CoA Quantifications.

For Acetyl CoA analyses, 30 mg third instar larvae from Oregon R and *ATPCL* mutants were homogenized and deproteinized using perchloric acid. Quantification of Acetyl-CoA was performed using the PicoProbe Acetyl CoA Fluorometric Assay kit (Biovision).

Chromosome cytology, immunostaining and microscopy.

DAPI-stained, colchicine-treated larval brain chromosome preparations for the analysis of chromosome aberration were made as previously described (Shaffer et al., 2006;Cipressa et al., 2016). Immunostaining was performed as previously described (Morciano et al., 2009). The primary antibodies and the dilutions used were as follows: anti-ATPCL (1:50), anti- α tubulin (1:2000; Sigma-Aldrich). A secondary antibody incubation was performed using both the FITC-conjugated anti-mouse IgG+IgM (1:20 in PBS; Jackson laboratories) and Alexa Fluor 488-conjugated anti-guinea pig IgG (1:300 in PBS; Jackson laboratories) for 2 h at room temperature. Slides were then mounted in Vectashield medium H-1200 with DAPI (Vector Laboratories) to stain DNA and reduce fluorescence fading.

Slides with mitotic chromosome preparations and fixed tissues were analyzed using a Zeiss Axioplan epifluorescence microscope (CarlZeiss, Obezkochen, Germany), equipped with a cooled CCD camera (Photometrics, Woburn, MA). Gray-scale digital images were collected separately, converted to Photoshop format, pseudocolored, and merged.

Rescue construct and germline transformation

For ATPCL rescue, a full length *ATPCL* encoding cDNA (LD21334, obtained from Drosophila Genomics Resource Center) was cloned into the *pUAST* plasmid under the control of a GAL4-promoter. Germline transformation with the resulting construct was carried out in *yw* lines by the Rainbow Transgenic Flies facility (Canarillo, CA) using standard procedures. A single insertion on chromosome 3 (*3[UAS-ATPCL; w+])* was selected for rescuing the *ATPCL* lethality. To this aim, *ATPCL/Sco; Tub-Gal4/MKRS* males were crossed to *ATPCL/CyO; 3[UAS-ATPCL; w+]/TM3* females and the resulting *ATPCL/ATPCL; Tub-Gal4/3[UAS-ATPCL; w+]* adults isolated.

Antibodies and Western blotting

To generate the anti-ATPCL antibody, a His-tag recombinant ATPCL protein fragment (Nterminal 1-350 aa) was expressed in *E. coli* and purified by SDS gel elettrophoresis The corresponding band was cut off the gel and sent to a specialized company (PRF&L Pocono Rabbit Farm and Laboratory, Canadensis, PA USA) for injection in guinea pig. Antibody specificity was checked first again the recombinant epitope expressed in *E. coli*. The anti serum recognizes specifically the fragment while the preimmune serum does not. To obtain extracts Western Blot analysis, total larvae were lysed in an ice-cold buffer containing 20 mM Hepes KOH pH 7.9,1.5 mM MgCl2, 10 mM KCl, 420 mM NaCl, 30 mM NaF,0.2 mM Na_3Vo_4, 25 mM BGP, 0.5 M PMSF, 0.1% NP40, 1_cprotease inhibitor cocktail (Roche). For immunoblotting, protein samples were resuspended in 1X Laemmli Buffer, run into SDS polyacrilammide gels and electroblotted on a nitrocellulose membrane (Bio-Rad) in a phosphate buffer containing 390 mM $NaH_2PO_4H_2O$ and 610 mM $Na_2HPO_42H_2O$. After blocking with 5% low-fat dry milk, the membrane was probed with appropriate primary antibody. The blots were developed by the ECL or ECL Plus method (Amersham Biosciences) and signals detected with the ChemiDoc scanning system (BioRad). The antibody dilutions were: anti-ATPCL (1:1000), anti-Tub (1:5000), anti AcH3 and anti acH4 (1:2000).

RNA extraction, cDNA amplification and qPCR

Total RNA was isolated from larval brains (50 brains/sample) using TRIzol (TRI Reagent® SIGMA Life Science). RNA concentration and purity were measured at the NanoDrop 1000 Spectrophotometer (ThermoScientific) with the NanoDrop 1000 3.7.1 software. Genomic DNA was eliminated with Invitrogen™ DNase I, Amplification Grade (ThermoFisher Scientific). To quantify the expression levels of *ATPCL* transcripts equal amounts of cDNA were synthesized with the iScript™ cDNA Synthesis Kit (BioRad). qPCR was performed using SsoAdvanced™ Universal SYBR® Green Supermix kit (BioRad) and conducted at 95°C for 10 min, and then 40 cycles of 95°C for 15 s and 60°C for 1 min. The specificity of the reaction was verified by melt curve analysis. 5pmol of both forward (5’-GTTGGGCTCCGGTTCCG -CAG, -ATG or -CTC -3’) and reverse (5’-ATTCCCCACACAATCGCCTT-3’) primers were used. RpL32 was amplified as the internal control. The threshold crossing value was noted for each transcript and normalized to the internal control. The relative quantitation of each mRNA was performed using the comparative Ct method. Experiments were performed using an ABI Prism 7300 System (Applied Biosystems), and data processing was performed using ABI SDS v2.1 software (Applied Biosystems).

RNA isolation, cDNA amplification and labeling for microarray analysis

Total RNA was isolated from wild-type (Oregon R) and mutant brains using the RNeasy Mini Kit (Qiagen). 50 ng of RNA were reverse transcribed and amplified using Access RT-PCR System kit (Promega). The cDNA was used as template for a two-step random PCR amplification; in Round A, Sequenase is used to extend randomly annealed primers (Primer A) to generate templates for subsequent PCR; during Round B, the specific primer B is used to amplify the templates previously generated and finally round C consists of additional PCR cycles to incorporate the amino allyl dUTP nucleotide. About 25 ng of each

cDNA sample was used for two 8 min extensions with 2.7 mM Round A primer (5′-GTT TCC CAG TCA CGA TCN NNN NNN NN-3′, N being a mixture of all four nucleotides with 60% A+T and 40% G+C) at 37°C with 267 U/ml Sequenase version 2.0 (usb). DNA was denatured at 94°C for 2 min and cooled to 10°C, and Sequenase 2.0 was added between extensions. The resulting products were used as template for 25 cycles of PCR using 1 U/100 µl Taq polymerase (Platinum Taq Invitrogen) and 10 mM Round B primer (5′-GTT TCC CAG TCA CGA TC-3′). Finally this DNA was used as template for 25 cycles of PCR to incorporate the amino allyl dUTP nucleotides to which the fluorescent dye may be attached (Randolph and Waggoner, 1997). To remove the Tris buffer which interferes with the indirect coupling, the aminoallyl-cDNA samples were desalted by filtering through a Microcon −30 and then mixed with the succinimidyl esters of the Cy3 or Cy5 dyes (Amersham Biosciences) in 0.1 M sodium bicarbonate buffer (pH 9); the coupling reaction was incubated overnight in the dark at room temperature. Each labelled sample was purified by AutoSeq MicroSpin G-50 columns (Amersham Biosciences) following the manufacturer's directions.

Microarray analysis

One µg of total RNA from each sample was linear amplified using the Ambion Amino Allyl MessageAmp™ II aRNA Amplification Kit (Life Technologies). Briefly, RNA was reverse transcribed using a primer containing both oligo(dT) and a T7 RNA polymerase promoter sequence. Double strand cDNA generated was used as template for in vitro transcription with T7 RNA polymerase. The modified nucleotide, 5-(3-aminoallyl)-UTP (aaUTP) was incorporated during the transcription step to produce amino allyl modified antisense RNA (aRNA). aaUTP was chemically coupled to reactive dyes (NHS ester dyes), Cy3 or Cy5 (GE Healthcare). Subsequently, slides were pre-hybridized at 50°C for 30 min in a solution containing 5× SSC, 0.1% SDS and 0.1% BSA. The labeled aRNAs (Cy3 sample and Cy5 sample mixed) were added to 50 µl of hybridization buffer containing 50% formamide, 10X SSC, 0.2% SDS pre-heated at 95°C for 3 min. Hybridization was carried out for 16 h at 42°C and unbound DNA was washed out using 3 steps with solutions containing: 1) 1X SSC 0.2% SDS pre-heated at 42°C; 2) 0.1X SSC 0.2 % SDS; 3) Two times 0.1X SSC.
A ScanArray Lite Microarray Scanner (Packard Bioscience) was used to acquire images, and GenePix Pro 6.1 software and ScanArray Express software were used to quantify hybridization signals. Absent and marginal spots were flagged automatically by software and subsequently each slide was inspected manually to eliminate artifacts, saturated spots, and low signal spots. Assuming that most of the genes have unchanged expression, the Cy5/Cy3 ratios were normalized within arrays using a Global Lowess Normalization and between arrays with the “Aquantile” method provided by the Bioconductor (Huber et al., 2015) package *limma* (Ritchie et al., 2015) running on R software (http://www.r-project.org). Genes significantly up- or down-regulated (2 fold cut-off and Bonferroni Hochberg corrected *P*-value < 0.1) were clustered by enrichment pathway analysis using Bioconductor R packages ClusterProfiler (Yu et al., 2012) using Gene Ontology Database annotations (Ashburner et al., 2000).

Supplementary Figures and Legends

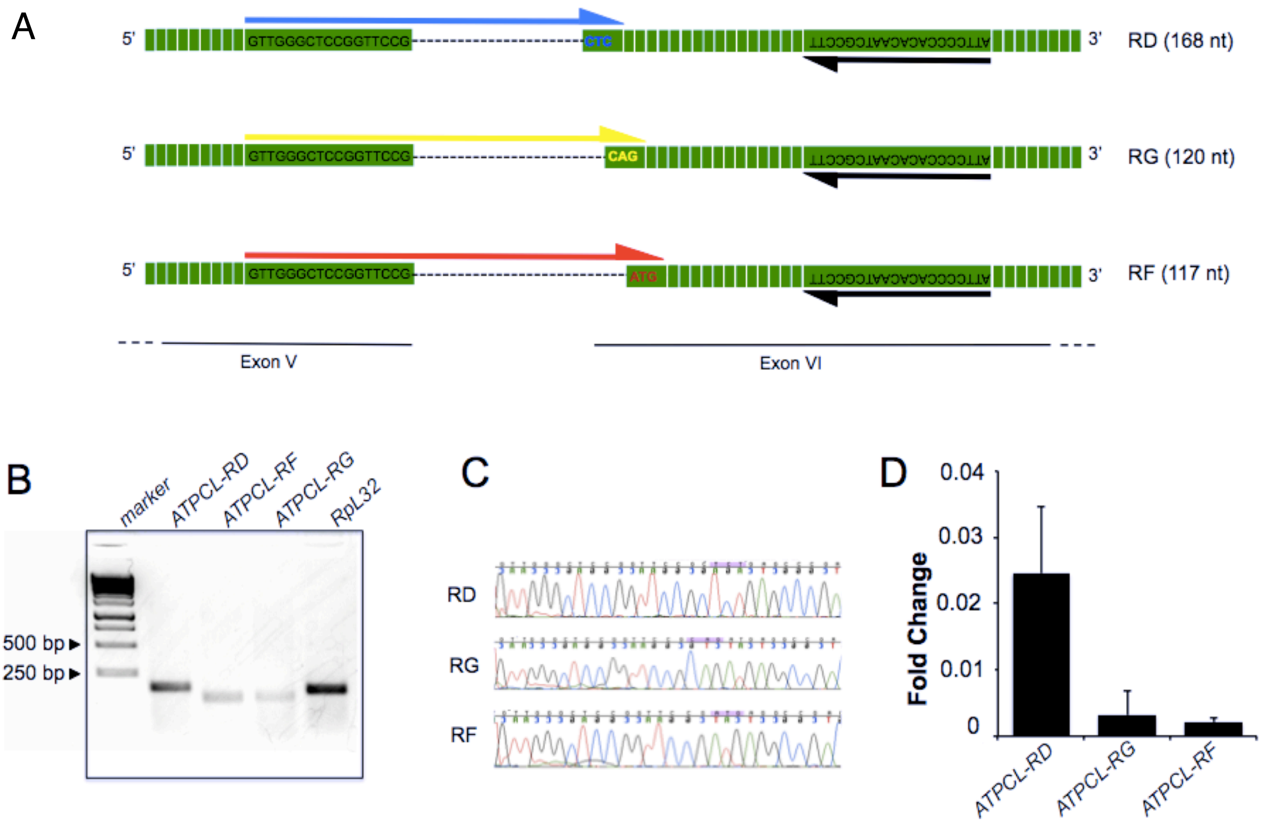

Supplementary Figure 1. Validation of *DmATPCL* transcripts.
Schematic representation of *DmATPCL* coding region including exon 5 (V) and exon 6 (VI) partial sequences used for primer designation. Colored arrows indicate *ad hoc* selected forward primers used in the RT-PCR experiment to discriminate exons 6 of *RD, RG* and *RE* thanks to the presence of the first 3bps (indicated in blue, yellow and red colors), which are unique for each transcript. The reverse primer (black arrow) is common for all transcripts. The expected sizes of each PCR product are indicated on the right. B) Agarose gel from RT-PCR analysis that shows different sized cDNA amplification products obtained with the same primers shown in A. Note the molecular weight are within the expected weight range shown in A. *Rpl32* has been used as the internal housekeeping control. C) Results of sequencing of PCR products shown in B. The electro-pherograms indicate the partial sequence of exon 5/exon 6 junction found in each PCR product including the 3 bps (highlighted in violet) which are specific for each transcript. D) Graph representing the expression levels of ATPCL transcripts revealed by qPCR on three different experiments. The fold change has been calculated using the comparative CT method with respect to *Rpl32.* Note that ATPCL-RD is about 8 times more abundant than RG and RF ($p<0.05$, Anova). Bars indicate standard deviation. See supplementary Text for details.

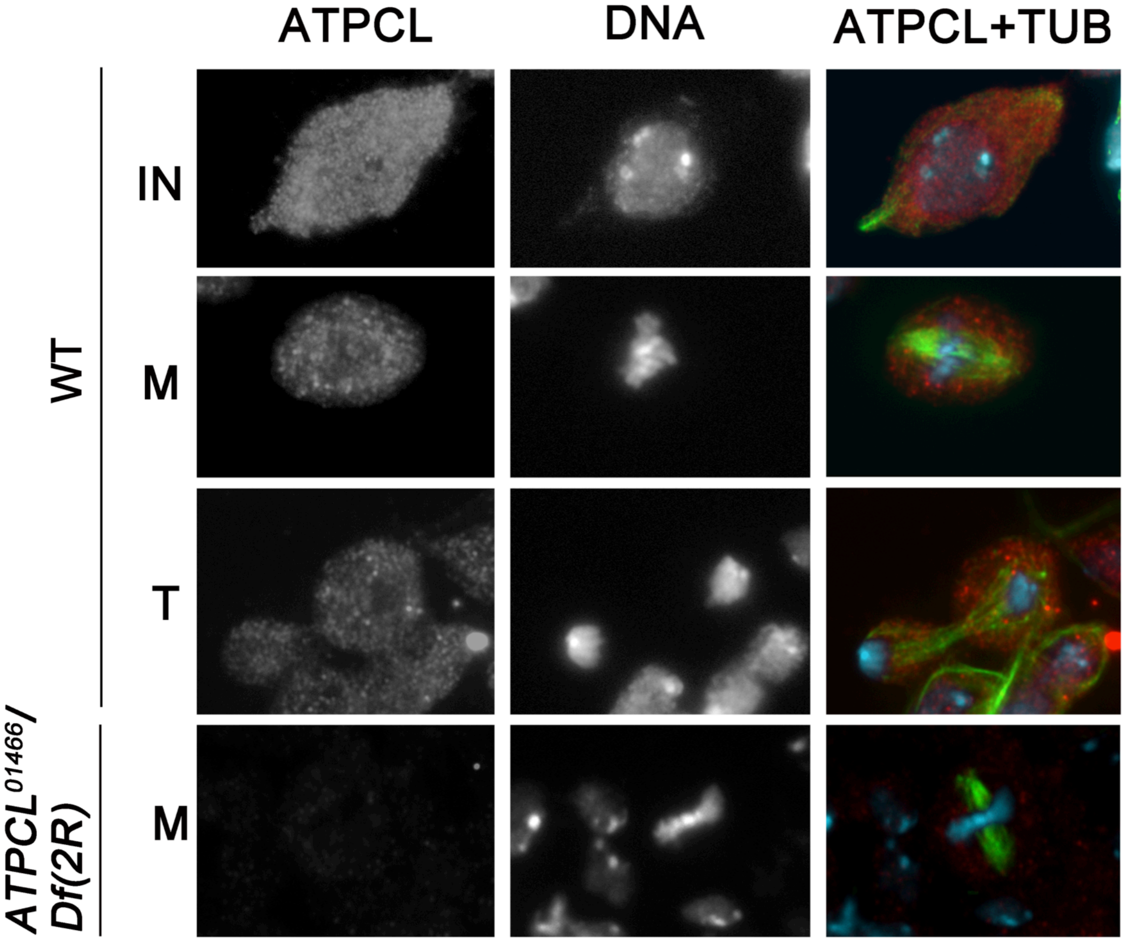

Supplementary Figure 2. Localization of ATPC in mitotic cells. A) anti-ATPCL immunostaining in control (OR/R) and *ATPCL*^{01466}*/ Df(2R)Exel7138* mutant mitotic cells. In interphase ATPCL localizes to both cytoplasm and nucleus while in dividing cells it is excluded from chromatin. Note that ATPCL localization is extremely reduced in the *ATPCL*^{01466}*/ Df(2R)Exel7138* mutant combination. IN: interphase; M: metaphase; T: telophase.

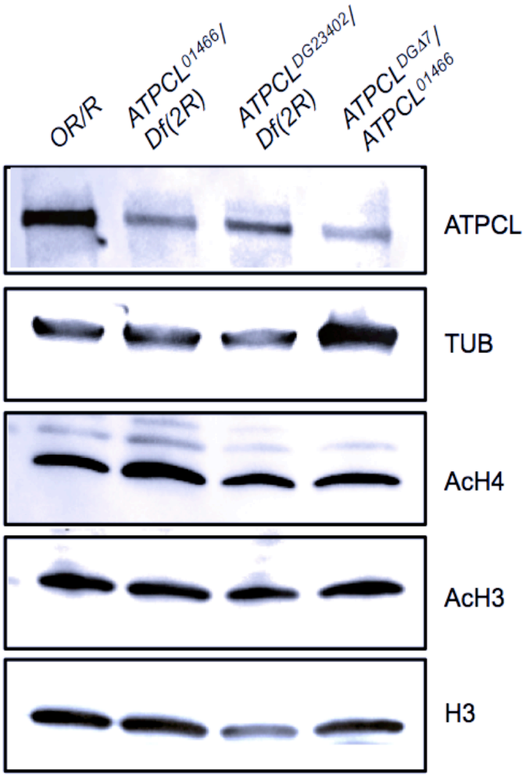

Supplementary Figure 3. Histone Acetylation in *DmATPCL* mutants. Western blot on larval brain extracts of different *DmATPCL* mutant combinations, that shows no significant difference in the levels of acetylated-H3 and –H4 between mutants and control OR/R. Both H3 and Tubulin have been used as loading controls.

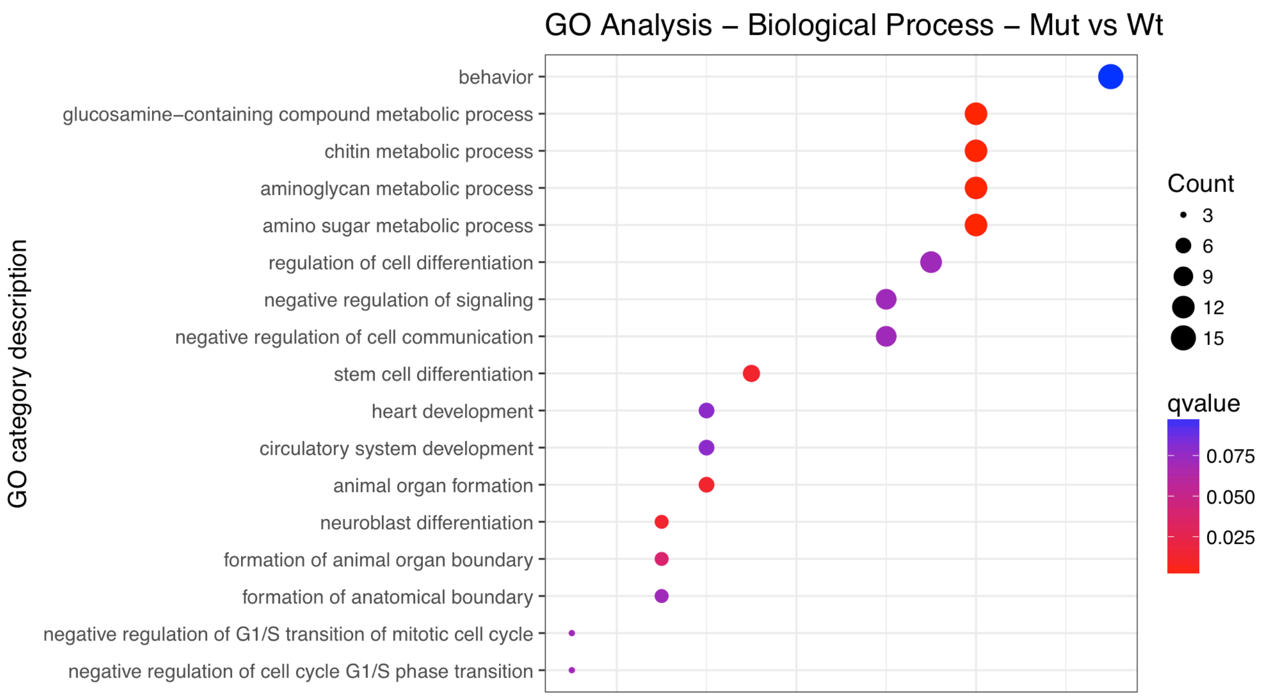

Supplementary Figure 4. Output of microarray analysis from *DmATPCL* mutants. Dotplot showing the results of enrichment analysis of modulated genes (2 fold cut-off and FDR < 0.1) using Gene Ontology database (Biological Process) annotation, in $ATPCL^{01466}$/ *Df(2R)Exel7138* mutant brains. The size of dots is correlated with number of genes that belong to a Gene Ontology category and dots are colored according the Benjamini-Hochberg false discovery rate adjusted p-values (qvalue) from blue (higher qvalue) to red (lower qvalue).

Supplementary Table 1. List of genes whose expression is modulated upon depletion of ATPCL. The table indicates the statistically significant differentially-expressed transcripts between $ATPCL^{01466}$/ *Df(2R)Exel7138* and wild-type brains. For each significantly-regulated (adjusted p-value < 0.1) transcript, microarray spot ID, official FlyBase number, gene symbol, textual gene definition, log2 Fold Change (*ATPCL* versus wt), transcript average expression, *P*-value and Bonferroni Hochberg corrected *P*-value is given.

Supplementary References

Cipressa, F., Morciano, P., Bosso, G., Mannini, L., Galati, A., Raffa, G.D., Cacchione, S., Musio, A., and Cenci, G. (2016). A role for Separase in telomere protection. *Nat Commun* 7**,** 10405.

Lattao, R., Bonaccorsi, S., Guan, X., Wasserman, S.A., and Gatti, M. (2011). Tubby-tagged balancers for the Drosophila X and second chromosomes. *Fly (Austin)* 5**,** 369-370.

Morciano, P., Carrisi, C., Capobianco, L., Mannini, L., Burgio, G., Cestra, G., De Benedetto, G.E., Corona, D.F., Musio, A., and Cenci, G. (2009). A conserved role for the mitochondrial citrate transporter Sea/SLC25A1 in the maintenance of chromosome integrity. *Hum Mol Genet* 18**,** 4180-4188.

Shaffer, C.D., Cenci, G., Thompson, B., Stephens, G.E., Slawson, E.E., Adu-Wusu, K., Gatti, M., and Elgin, S.C. (2006). The large isoform of Drosophila melanogaster heterochromatin protein 2 plays a critical role in gene silencing and chromosome structure. *Genetics* 174**,** 1189-1204.
